# PESV represses non-small cell lung cancer cell malignancy through circ_0016760 under hypoxia

**DOI:** 10.1186/s12935-021-02336-6

**Published:** 2021-11-27

**Authors:** Hong Zhang, Haojian Zhang, Jiye Zhu, Huan Liu, Qin Zhou

**Affiliations:** Department of Oncology, The First Hospital of Hunan University of Chinese Medicine, No.95 Shaoshan Middle Road, Yuhua District, Changsha, 410007 Hunan China

**Keywords:** PESV, circ_0016760, miR-29b, HIF1A, NSCLC

## Abstract

**Background:**

Non-small cell lung cancer (NSCLC) accounts for more than 80% of lung cancers, which is the most common malignant tumor worldwide. Polypeptide extract from scorpion venom (PESV) has been reported to inhibit NSCLC process. The present study aims to reveal the roles of PESV in NSCLC progression under hypoxia and the inner mechanism.

**Methods:**

The expression levels of circular RNA 0016760 (circ_0016760) and microRNA-29b (miR-29b) were detected by quantitative real-time polymerase chain reaction (qRT-PCR). Protein expression was determined by western blot and immunohistochemistry assays. Cell migration, invasion, proliferation and tube formation were investigated by transwell, cell colony formation, 3-(4,5-Dimethylthazol-2-yl)-2,5-diphenyltetrazolium bromide and tube formation assays. The impacts between PESV and circ_0016760 overexpression on tumor growth in vivo were investigated by in vivo tumor formation assay.

**Results:**

Circ_0016760 expression was dramatically upregulated in NSCLC tissues and cells, compared with adjacent lung tissues and cells, respectively. PESV treatment downregulated circ_0016760 expression. Circ_0016760 silencing or PESV treatment repressed cell migration, invasion, proliferation and tube formation under hypoxia in NSCLC cells. Circ_0016760 overexpression restored the effects of PESV treatment on NSCLC process under hypoxia. Additionally, circ_0016760 acted as a sponge of miR-29b, and miR-29b bound to HIF1A. Meanwhile, miR-29b inhibitor impaired the influences of circ_0016760 knockdown on NSCLC process under hypoxia. Further, ectopic circ_0016760 expression restrained the effects of PESV exposure on tumor formation in vivo.

**Conclusion:**

Circ_0016760 overexpression counteracted PESV-induced repression of NSCLC cell malignancy and angiogenesis under hypoxia through miR-29b/HIF1A axis.

**Supplementary Information:**

The online version contains supplementary material available at 10.1186/s12935-021-02336-6.

## Introduction

Lung cancer-related mortality accounts for more than 15% of cancer-caused deaths worldwide [1]. Non-small cell lung cancer (NSCLC) accounts for more than four fifths of total lung cancers, posing a growing threat to human health [2, 3]. Hypoxia is a common pathological phenomenon in cancer development and can facilitate tumor growth [4]. Under hypoxia, glycolysis provides energy for cancer cell proliferation and motility [5]. Scorpion can promote blood circulation and remove blood stasis, and polypeptide extract from scorpion venom (PESV) is able to inhibit cell proliferation in cancers, including NSCLC [6, 7]. However, the mechanism of PESV in regulating NSCLC progression under hypoxia is still unclear.

Circular RNA (circRNA) belongs to the members of endogenous RNAs with neither 5’ caps nor 3’ polyadenylated tails [8]. CircRNA is involved in the pathogenesis of NSCLC. For example, circ_0074027 overexpression promotes cell proliferation and metastasis via sponging microRNA-185-3p (miR-185-3p) [9]. On the contrary, enforced circ_0078767 expression hinders cell proliferation and invasion through interaction with miR-330-3p [10]. Additionally, circ_0016760 promotes cell proliferation and metastasis via associating with miR-1287 and miR-577 in NSCLC [11, 12]. Nevertheless, whether circ_0016760 is involved in PESV-mediated NSCLC progression under hypoxia is unknown.

MiRNA is a noncoding RNA that acts function via binding to target gene, leading to target gene degradation or protein inhibition [13]. Previous evidence has suggested that miRNA participates in cancer progression [14]. MiR-29b, a cancer-related miRNA, commonly acts as a tumor suppressor [15]. In NSCLC, miR-29b has been reported to repress cell proliferation, migration and invasion [16, 17], implicating that miR-29b may be helpful to develop the therapeutic strategy of NSCLC. In a low hypoxia condition, hypoxic inducible family of transcription factors (HIF) regulates some biological processes related to cell growth [18]. HIF1A is a vital regulator in hypoxic response and can modulate hypoxic gene expression [19, 20]. It is activated by prolyl 4-hydroxylases under hypoxia after the reduction of hydroxylation [21]. It has been found that HIF1A combines with miR-199a to inhibit NSCLC cell proliferation under hypoxia [22]. Wang et al. also suggested that miR-182 promoted glycolysis by increasing HIF1A expression [23]. These evidences demonstrate the importance of HIF1A in the progression of NSCLC. As predicted by online databases, miR-29b contained the potential complementary sites of circ_0016760 and HIF1A.

Thus, the present study explored the impacts of circ_0016760 depletion on NSCLC malignant development and tube formation under hypoxia, and determined whether the inner mechanism responsible for the effects of circ_0016760 on PESV-mediated NSCLC malignant progression involved circ_0016760/miR-29b/HIF1A axis. Herein, we detected circ_0016760 expression in PESV-treated NSCLC cells, analyzed the impacts of circ_0016760 on malignant development and tube formation of NSCLC cells treated with PESV under hypoxia, and determined whether PESV-mediated NSCLC cell processes was associated with circ_0016760/miR-29b/HIF1A axis.

## Materials and methods

### Clinical specimen and cell culture

Human NSCLC tissues and paracancerous normal lung tissues (N = 43, respectively) were collected from NSCLC patients from the First Hospital of Hunan University of Chinese Medicine. Tissues were restored at − 80 °C. The Ethics Committee of the First Hospital of Hunan University of Chinese Medicine approved this research. The participants signed the written informed consents. The clinicopathologic features of 43 NSCLC patients were shown in Additional file 1: Table S1.

Human lung epithelial cell-line BEAS-2B, NSCLC cell lines (A549 and H1299) and human umbilical vein endothelial cells (HUVEC) were acquired from Procell (Wuhan, China). Cells were cultured in Roswell Park Memorial Institute-1640 medium (RPMI-1640; Procell), bronchial epithelial cell basal medium (BEBM; Procell) or Ham’s F12K (Procell) at 37 °C with 95% air and 5% CO_2._ Medium was supplemented with 10% fetal bovine serum (FBS; Procell) as well as antibiotics (100 μg/mL penicillin, 100 μg/mL streptomycin) (Millipore, Bradford, MA, USA). For hypoxia treatment, cells were cultured in a hypoxia incubator chamber (Maworde, Qiqihar, China) with 5% CO_2_, 1% O_2_ and 94% N_2_. PESV, obtained from Chinese Medicine Pharmacy of the First Affiliated Hospital of Hunan University of Chinese Medicine, was used to study the role of circ_0016760 in PESV-mediated NSCLC development and the underlying mechanism.

### Cell transfection

Ribobio (Guangzhou, China) provided the small interfering RNAs against circ_0016760 (si-circ_0016760^#1^, 5’-GTCTGGCATGCAGAGGCAGAA-3’; si-circ_0016760^#2^, 5’-CTGGCATGCAGAGGCAGAAGA-3’ and si-circ_0016760^#3^, 5’-ATGCAGAGGCAGAAGAGGCCT-3’), the small hairpin RNA targeting circ_0016760 (sh-circ_0016760), miR-29b mimic (miR-29b, 5’-UAGCACCAUUUGAAAUCAGUGUU-3’), miR-29b inhibitor (in-miR-29b, 5’-AACACUGAUUUCAAAUGGUGCUA-3’) and controls (si-con, con, miR-con and in-miR-con). The plasmids overexpressing circ_0016760 (circ_0016760) and HIF1A (HIF1A) were built in Geneseed (Guangzhou, China) using pCD5-ciR and pcDNA vectors.

Si-circ_0016760^#2^ and circ_0016760 were transfected into cells with controls to reveal the effects of circ_0016760 on PESV-mediated NSCLC process under hypoxia. Si-circ_0016760^#2^, in-miR-29b, miR-29b or HIF1A was employed to determine the relationships among circ_0016760, miR-29b and HIF1A in regulating NSCLC cell processes under hypoxia. sh-circ_0016760, circ_0016760 and con were employed to demonstrate the impacts between circ_0016760 and PESV treatment on tumor growth in vivo. Cell transfection was conducted using Lipofectamine 2000 (Thermo Fisher, Waltham, MA, USA) according to the manufacturer’s instructions.

### Quantitative real-time PCR (qRT-PCR)

TsingZol (Tsingke, Shanghai, China) was used to lyse tissues and cells. RNA isolation reagents (Corning, Madison, New York, USA) were then employed to isolate RNA. Reverse transcription was performed with High-Capacity cDNA Synthesis kits (Thermo Fisher). For detecting the amount of circRNA/miRNA/mRNA, Fast qPCR Mix (Tsingke) was employed. Data were assessed using the 2^−∆∆Ct^ method with U6 and β-actin as controls. Additionally, oligo(dT)18 primers and random primers (Solarbio, Beijing, China) were employed to confirm the circular structure of circRNA. The sense and antisense primers were circ_0016760 5’-AGAGGTTATCCCCATTTTAGAAGTG-3’ and 5’-CATCTGTTCCTGGGTCTGT-3’; HIF1A 5’-GTCTGAGGGGACAGGAGGAT-3’ and 5’-AAAGGCAAGTCCAGAGGTGG-3’; U6 5’-CTCGCTTCGGCAGCACA-3’ and 5’-AACGCTTCACGAATTTGCGT-3’; β-actin 5’-CACCATTGGCAATGAGCGGTTC-3’ and 5’-AGGTCTTTGCGGATGTCCACGT-3’. Ribobio Co., Ltd. provided the primers for miR-29b.

### Transwell assay

Cell migration and invasion were analyzed by transwell chambers. In short, A549 cells and H1299 cells were mixed with serum-free Ham’s F12K medium (Procell) or RPMI-1640 medium (Procell), and added into the upper chamber, which was coated with Matrigel (Corning) for invasion assay. Ham’s F12K medium and RPMI-1640 medium with 15% FBS (Procell) were severally placed into the lower chamber. At 24 h after transfection of plasmids and oligonucleotides, medium was removed and cells were incubated with methanol (Beyotime, Shanghai, China) and crystal violet (Beyotime). Results were demonstrated via calculating the number of cells from 6 high-power (100x) field microscope (Olympus, Tokyo, Japan).

### Cell colony formation assay

The colony-forming ability of A549 and H1299 cells was revealed by colony formation assay. Shortly, cells (500 cells per well) were grown in 6-well plates for 2 weeks after transfection of plasmids and oligonucleotides, and Ham’s F12K medium and RPMI-1640 medium (Procell) were replaced every 3 days. Proliferative colonies were immobilized and stained with paraformaldehyde (Beyotime) and crystal violet (Beyotime), respectively. Cell colony-forming ability was analyzed by determining the number of colonies.

### 3-(4,5-Dimethylthazol-2-yl)-2,5-diphenyltetrazolium bromide (MTT) assay

Cells were grown in 96-well plates for 16 h, and treated with test compounds. At 48 h after transfection, cells were went through 4 h incubation with MTT solution (Beyotime). After that, dimethyl sulfoxide was placed into each well. Samples were assessed via detecting the absorbance at 490 nm with microplate reader (REAGEN, Shenzhen, China).

### Tube formation assay

Angiogenic capacity of cells was analyzed by capillary-like network formation assay as descripted previously [24]. In brief, the cells were passaged in 96-well microplates coated with growth factor-depleted Matrigel (Corning). After 16 h of culture in NSCLC-conditioned medium, the branch points containing at least 3 cells were counted under microscope (Zeiss, Melville, NY, USA). Results were analyzed using image J software.

### Flow cytometry analysis

Cell apoptotic rate was analyzed using Annexin V-FITC and propidium iodide apoptotic detection kit (Solarbio). In brief, the cells were harvested after various treatments, and suspended in Binding buffer. Then, Annexin V-FITC and propidium iodide were used to incubate the cells in the dark. Finally, cell apoptotic rate was analyzed using a flow cytometer with CytExpert software.

### Dual-luciferase reporter assay

The binding sequences between miR-29b and circ_0016760 or HIF1A were predicted by starbase online database and microT CDS online database, respectively. The wild-type (WT) plasmids of circ_0016760 (circ_0016760-WT) and HIF1A (HIF1A-WT) and their mutant (MUT) plasmids (circ_0016760-MUT and HIF1A-MUT) were constructed by Ribobio Co., Ltd. Cell transfection was performed according to the literature’s methods [25]. Luciferase activities were detected using a Dual-Lucy Assay Kit (Solarbio).

### RNA immunoprecipitation (RIP) assay

In short, cells were harvested and lysed using RIP lysis buffer (Millipore). Lysates were incubated with magnetic beads coated with anti-argonaute2 (anti-Ago2; Abcam, Cambridge, UK) or anti-immunoglobulin G (anti-IgG; Abcam) for 24 h. The expression of circ_0016760, miR-29b and HIF1A enriched by anti-Ago2 or anti-IgG was detected by qRT-PCR.

### Western blot analysis

The lysates from cells and tissues were acquired by using RIPA buffer (Beyotime), and then loaded onto 12% SurePAGE gels (Thermo Fisher). Protein bands were electrotransferred onto polyvinylidene fluoride membranes (Millipore) prior to blocking aspecific signals using 5% nonfat milk (Solarbio). After that, the membranes were incubated with primary antibodies and secondary antibody (1:8000; Affinity, Nanjing, China), respectively. Protein bands were visualized using RapidStep ECL Reagent (Millipore). β-actin was employed as a reference. Primary antibodies were anti-HIF1A (1:1500; Affinity), anti-proliferating cell nuclear antigen (anti-PCNA; 1:800; Otwo Biotech, Shenzhen, China) and anti-β-actin (1:1000; CST, Boston, MA, USA).

### In vivo tumor formation assay

Vital River Laboratories (Beijing, China) provided male BALB/c nude mice (5-week old). Nude mice were divided into 4 groups (N = 6, respectively): con group, sh-circ_0016760 group, PESV group and PESV + circ_0016760 group. 5 × 10^6^ A549 cells transfected with sh-circ_0016760, con or circ_0016760 were hypodermically injected into the mice. Mice were intragastrically administered with PESV (200 mg/kg) once a day from tumor formation to the end of experiments. On the 7th day after injection, tumor volume was monitored every 7 days. Twenty-eight days later, mice were administrated with xylazine (10 mg/kg; Seebio Biotech, Shanghai, China) and then euthanized by cervical dislocation. The forming tumors were excised for the analysis of tumor weight and gene expression. The Animal Care Committee of the First Hospital of Hunan University of Chinese Medicine agreed with this study.

### Immunohistochemistry (IHC) assay

IHC assay was performed on the primary tumors from A549 cells according to the standard method [26]. In brief, 4-µm-thick sections embedded into paraffin were heated and deparaffinized with xylene. The slides were incubated with H_2_O_2_ and primary antibodies specific to HIF1A (1:200; Cusabio Biotech, Wuhan, China), proliferating cell nuclear antigen (Ki67; 1:200; Cusabio Biotech) and Cleaved Caspase-3 (1:200; Cusabio Biotech). Subsequent steps were carried out using IHC assay kit (Phygene, Fuzhou, China) as instructed. CX31-LV320 microscope (Olympus) was utilized to capture images. The relative expression of the three proteins was calculated based on the percentage of stained cells and intensity of immunostaining as instructed [27].

### Statistical analysis

SPSS 21.0 software was employed to analyze the data from 3 independent duplicate tests. Results were shown as means ± standard deviations (SD). Significant differences were demonstrated by Student’s *t*-tests, Wilcoxon rank-sum test or one-way analysis of variance. *P* value < 0.05 was considered statistically significant.

## Results

### PESV treatment downregulated circ_0016760 expression in A549 and H1299 cells

**Fig. 1:**
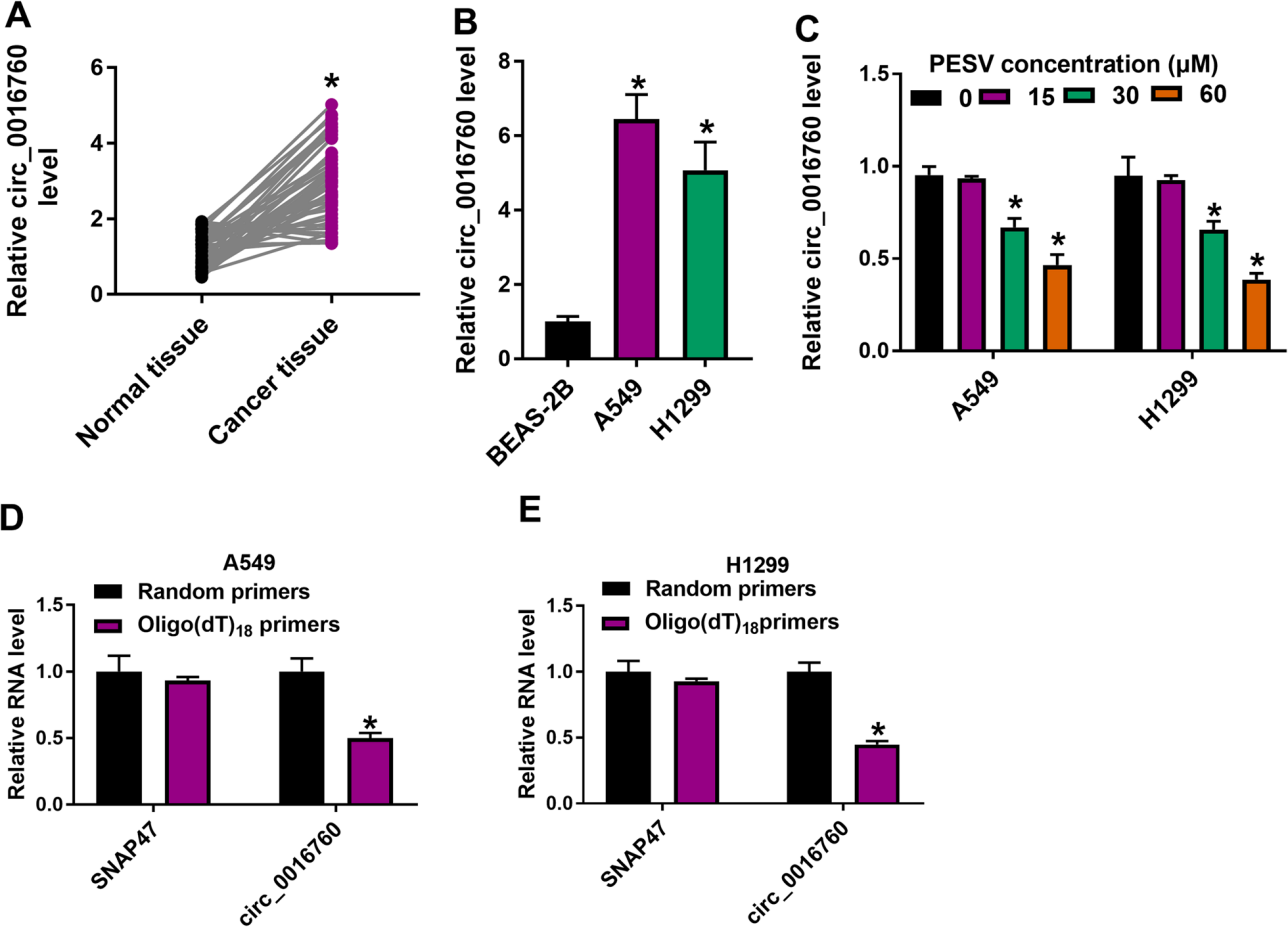
Circ_0016760 expression was decreased after PESV exposure in NSCLC cells. **A**, **B** Circ_0016760 expression was detected by qRT-PCR in NSCLC tissues (N = 43), paracancerous normal tissues (N = 43) as well as BEAS-2B, A549 and H1299 cells. **C** Circ_0016760 expression was detected at 24 h after PESV treatment (0, 15, 30 and 60 μM) by qRT-PCR in A549 and H1299 cells. **D**, **E** The circular structure of circ_0016760 was identified by using oligo(dT)_18_ primers and random primers. *P < 0.05

To determine whether PESV-mediated NSCLC progression involved circ_0016760, we first detected circ_0016760 expression in NSCLC tissues and cells. Results showed that circ_0016760 expression was dramatically upregulated in NSCLC tissues and A549 and H1299 cells compared with paracancerous normal lung tissues and BEAS-2B cells, respectively (Fig. 1A, B). Subsequently, the study analyzed the effects of PESV treatment on circ_0016760 expression in A549 and H1299 cells. qRT-PCR data showed that circ_0016760 expression was decreased in the both A549 and H1299 cells after PESV treatment (30 μM and 60 μM), especially in 60 μM PESV-treated NSCLC cells (Fig. 1C). Therefore, 60 μM PESV was employed for further study. Besides, we identified the stability of circ_0016760 using oligo(dT)_18_ and random primers. As presented in Fig. 1D, E, oligo(dT)_18_ primers significantly amplified linear SNAP47 rather than circ_0016760, which suggested that circ_0016760 lacked poly (A) tails. These data demonstrated that PESV could regulate circ_0016760 expression in NSCLC cells.

### Circ_0016760 silencing attenuated hypoxia-induced cell migration, invasion, proliferation and tube formation in A549 and H1299 cells

**Fig. 2:**
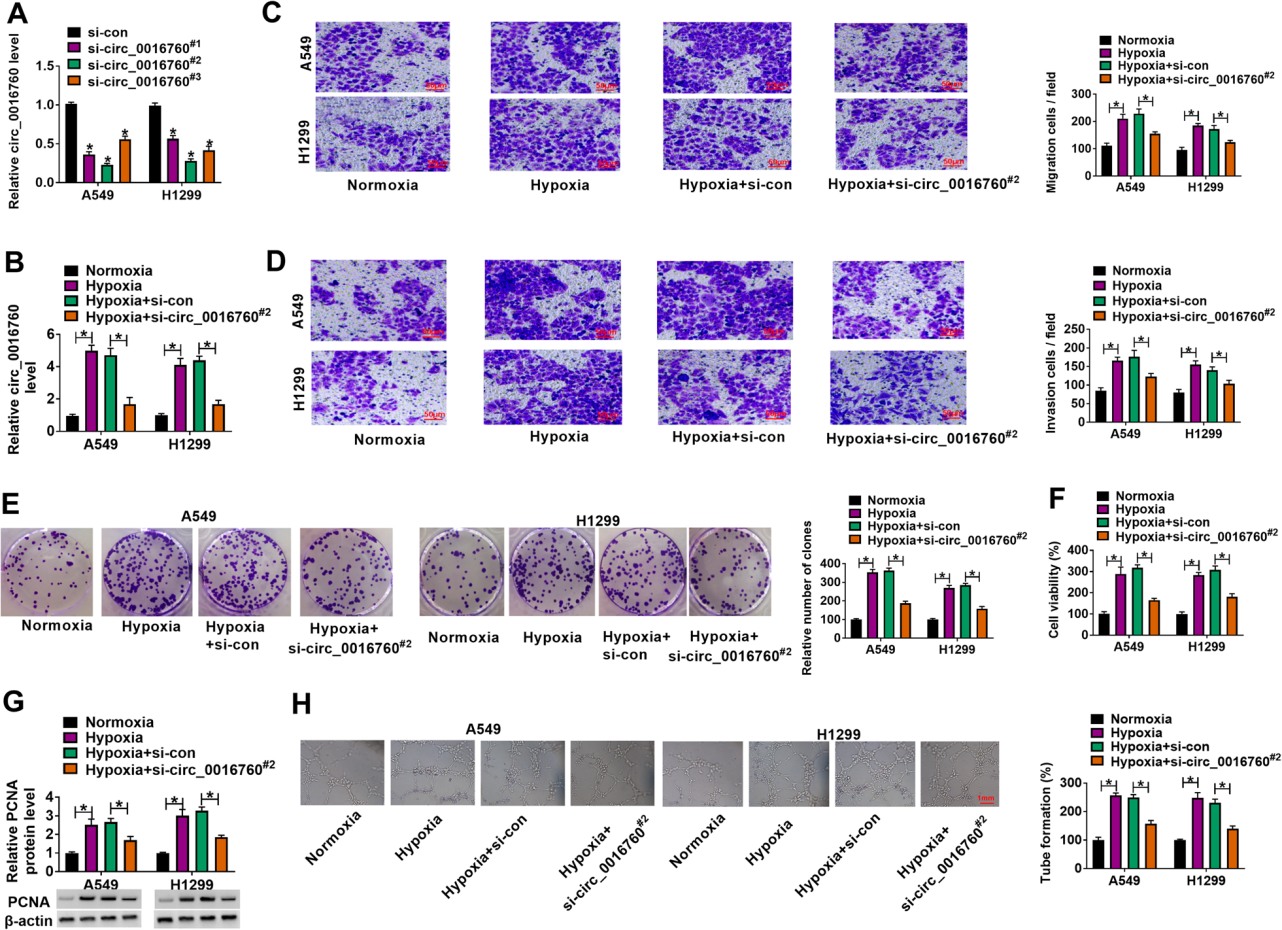
Circ_0016760 knockdown partly abolished the impacts of hypoxia treatment on NSCLC cell malignancy. **A** Circ_0016760 expression was determined by qRT-PCR in A549 and H1299 cells transfected with si-con, si-circ_0016760^**#**1^, si-circ_0016760^**#**2^ or si-circ_0016760^**#**3^. **B** The influence of circ_0016760 knockdown on circ_0016760 expression under hypoxia was detected by qRT-PCR in A549 and H1299 cells. **C**, **D** The impacts of circ_0016760 repression on the migration and invasion of A549 and H1299 cells after hypoxia treatment were demonstrated by transwell assay. **E**, **F** Cell colony formation and MTT assays were employed to investigate the effect of circ_0016760 knockdown on the proliferation of A549 and H1299 cells under hypoxia. (**G**) The effect of circ_0016760 silencing on PCNA expression under hypoxia was determined by western blot. **H** Tube formation assay was performed to reveal the impact of circ_0016760 knockdown on tube formation under hypoxia. *P < 0.05

In order to demonstrate the impacts of circ_0016760 silencing on NSCLC process under hypoxia, the efficiency of si-circ_0016760^#1^, si-circ_0016760^#2^ and si-circ_0016760^#3^ in interfering circ_0016760 expression was firstly determined. Results showed that circ_0016760 expression was significantly downregulated by si-circ_0016760^#1^, si-circ_0016760^#2^ and si-circ_0016760^#3^, especially by si-circ_0016760^#2^ (Fig. 2A). Based on the consequence, si-circ_0016760^#2^ was chosen for further experiments. Subsequently, we transfected the small interfering RNA of circ_0016760 into hypoxia-induced A549 and H1299 cells to determine the consequential effects. qRT-PCR results showed that hypoxia treatment upregulated circ_0016760 expression, whereas circ_0016760 silencing impaired this effect (Fig. 2B). Transwell assay displayed that hypoxia treatment promoted cell migration and invasion, whereas these impacts were restored after circ_0016760 knockdown (Fig. 2C, D). Similarly, the colony-forming ability and viability of A549 and H1299 cells were also facilitated under hypoxia; however, these influences were partially abolished by reduced expression of circ_0016760 (Fig. 2E, F). In support, hypoxia-induced upregulation of PCNA expression was remitted after circ_0016760 knockdown (Fig. 2G). Further, hypoxia treatment promoted tube formation, which was rescued after knockdown of circ_0016760 (Fig. 2H). Thus, these results demonstrated that circ_0016760 silencing could attenuate hypoxia-induced the migration, invasion, proliferation and tube formation of NSCLC cells.

### Circ_0016760 overexpression restrained the inhibitive effects of PESV treatment on NSCLC process under hypoxia

**Fig. 3:**
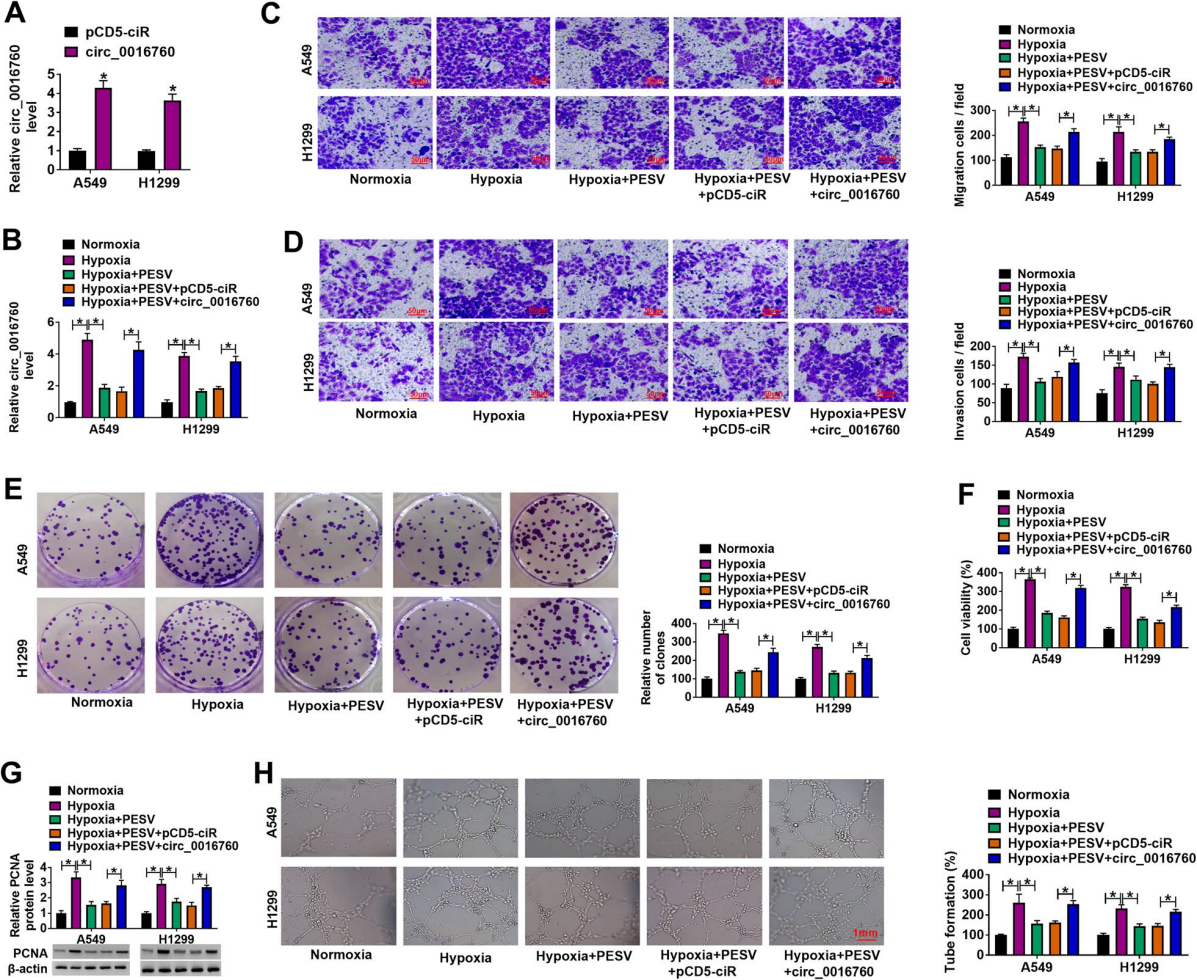
Circ_0016760 overexpression attenuated the impacts of PESV treatment on NSCLC cell processes under hypoxia. **A** The overexpression efficiency of circ_0016760 was detected by qRT-PCR in A549 and H1299 cells. **B** The effects between PESV treatment and circ_0016760 overexpression on circ_0016760 expression under hypoxia were determined by qRT-PCR in A549 and H1299 cells. **C**, **D** Transwell assay was conducted to determine the influences between PESV treatment and circ_0016760 overexpression on the migration and invasion of A549 and H1299 cells under hypoxia. **E**, **F** Cell colony formation and MTT assays were carried out to unveil the influences between PESV treatment and enforced circ_0016760 expression on cell proliferation under hypoxia in A549 and H1299 cells. **G** Western blot analysis was carried out to determine the influences between PESV treatment and circ_0016760 overexpression on PCNA protein expression under hypoxia. **H** Tube formation assay was conducted to determine the influences between PESV treatment and circ_0016760 overexpression on tube formation under hypoxia. *P < 0.05

Based on the above results, the study continued to analyze the effects between PESV treatment and circ_0016760 on NSCLC process under hypoxia. Results firstly showed that PESV treatment induced A549 and H1299 cell apoptosis but had no effect on BEAS-2B cells (Additional file 2: Fig. S1). Circ_0016760 expression was upregulated after transfection with circ_0016760 (Fig. 3A), suggesting that circ_0016760 was effective in increasing circ_0016760 expression. Subsequently, results showed that PESV exposure reversed the promoting effect of hypoxia treatment on circ_0016760 expression; however, this impact was partly abolished after circ_0016760 overexpression (Fig. 3B). Besides, we found that PESV treatment hindered hypoxia-induced cell migration, invasion and proliferation, whereas these impacts were restrained after circ_0016760 overexpression (Fig. 3C–F). PESV exposure also attenuated hypoxia-induced PCNA expression, but ectopic circ_0016760 expression impaired this influence (Fig. 3G). Comparatively, PESV inhibited tube formation under normoxia, whereas these effects were attenuated by ectopic circ_0016760 expression (Fig. 3H). These results explained that PESV could repress NSCLC cell migration, invasion, proliferation and tube formation under hypoxia by regulating circ_0016760.

### Circ_0016760 upregulated HIF1A expression by sponging miR-29b

**Fig. 4:**
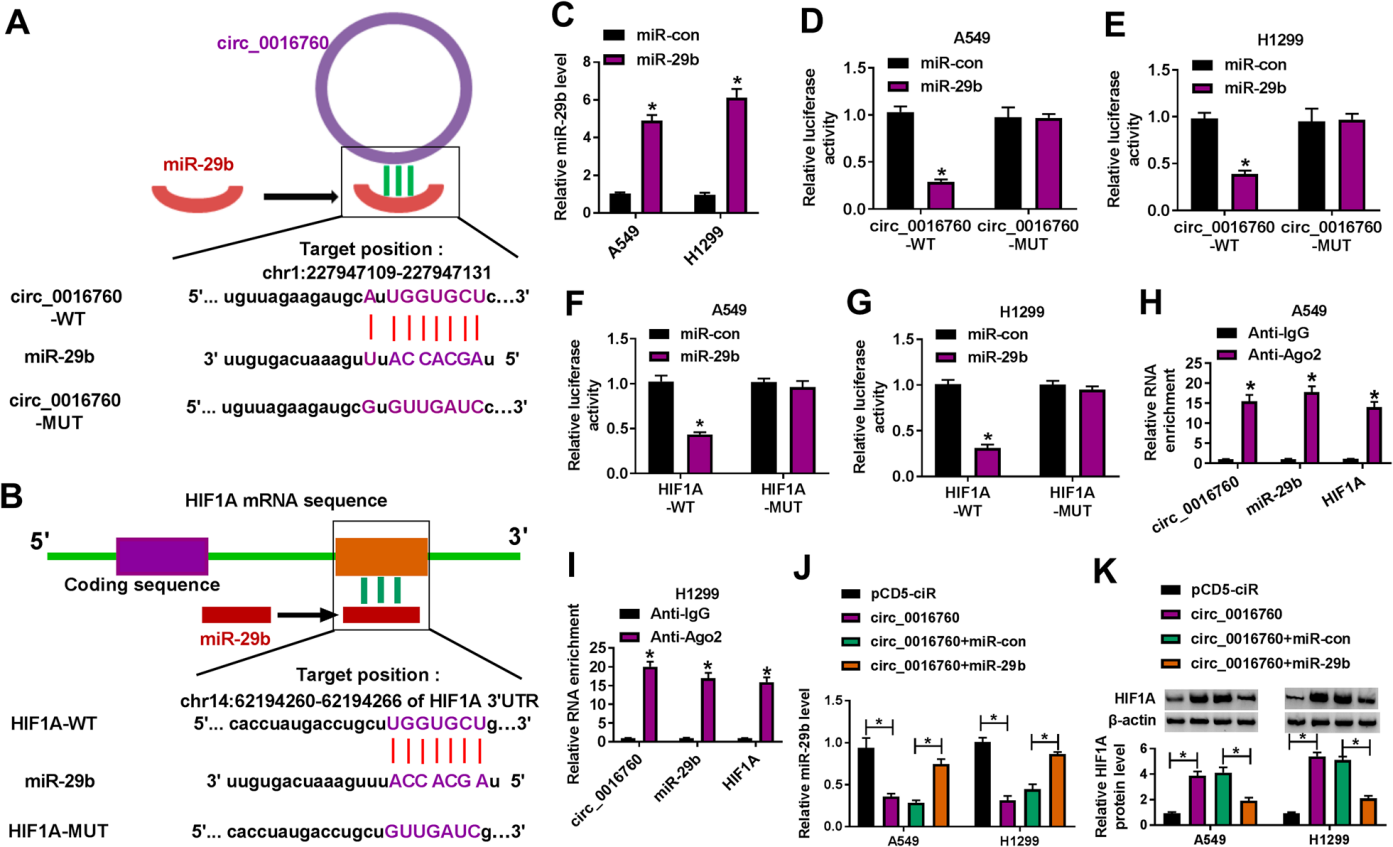
Circ_0016760 regulated HIF1A expression via sponging miR-29b in NSCLC cells. **A** Starbase online database was employed to predict the putative binding sites between miR-29b and circ_0016760. **B** MicroT-CDS online database was performed to predict the putative binding sequence between miR-29b and HIF1A. **C** MiR-29b expression was detected by qRT-PCR in A549 and H1299 cells transfected with miR-29b or miR-con. **D**–**G** Dual-luciferase reporter assay was conducted to detect luciferase activity in A549 and H1299 cells. **H**, **I** RIP assay was carried out to reveal the direct binding relationship between miR-29b and circ_0016760 or HIF1A. **J**, **K** The effects between ectopic circ_0016760 expression and miR-29b mimic on miR-29b expression and HIF1A protein expression were demonstrated by qRT-PCR and western blot, respectively, in A549 and H1299 cells. *P < 0.05

Considering the feasibility of circRNA-miRNA-mRNA network in revealing the pathogenesis of cancers, the miRNA and mRNA related to circ_0016760 were further analyzed. As presented in Fig. 4A, B, circ_0016760 contained the binding sites of miR-29b, and miR-29b possessed the binding sequence of HIF1A. To identify the binding relationship between miR-29b and circ_0016760 or HIF1A, we detected the efficiency of miR-29b overexpression (Fig. 4C). Dual-luciferase reporter assay showed that the luciferase activity was dramatically repressed in miR-29b mimic and circ_0016760-WT group as well as in miR-29b mimic and HIF1A-WT group, but not in miR-29b mimic and circ_0016760-MUT group or in miR-29b mimic and HIF1A-MUT group (Fig. 4D–G). Meanwhile, RIP assay showed that circ_0016760, miR-29b and HIF1A could be enriched in anti-Ago2 groups as compared to anti-IgG group (Fig. 4H, I). Further, results displayed that circ_0016760 overexpression dramatically downregulated miR-29b expression and upregulated HIF1A protein expression, whereas miR-29b mimic attenuated these effects (Fig. 4J, K). The similarly results were also observed in PESV-treated A549 and H1299 cells (Additional file 3: Fig. S2). For instance, increasing circ_0016760 expression decreased miR-29b expression but increased HIF1A expression in PESV-treated A549 and H1299 cells, which were relieved after miR-29b introduction. The above data demonstrated that circ_0016760 induced HIF1A production through interaction with miR-29b in NSCLC cells.

### Circ_0016760 silencing inhibited cell migration, invasion, proliferation and angiogenesis by sponging miR-29b under hypoxia in NSCLC cells

**Fig. 5:**
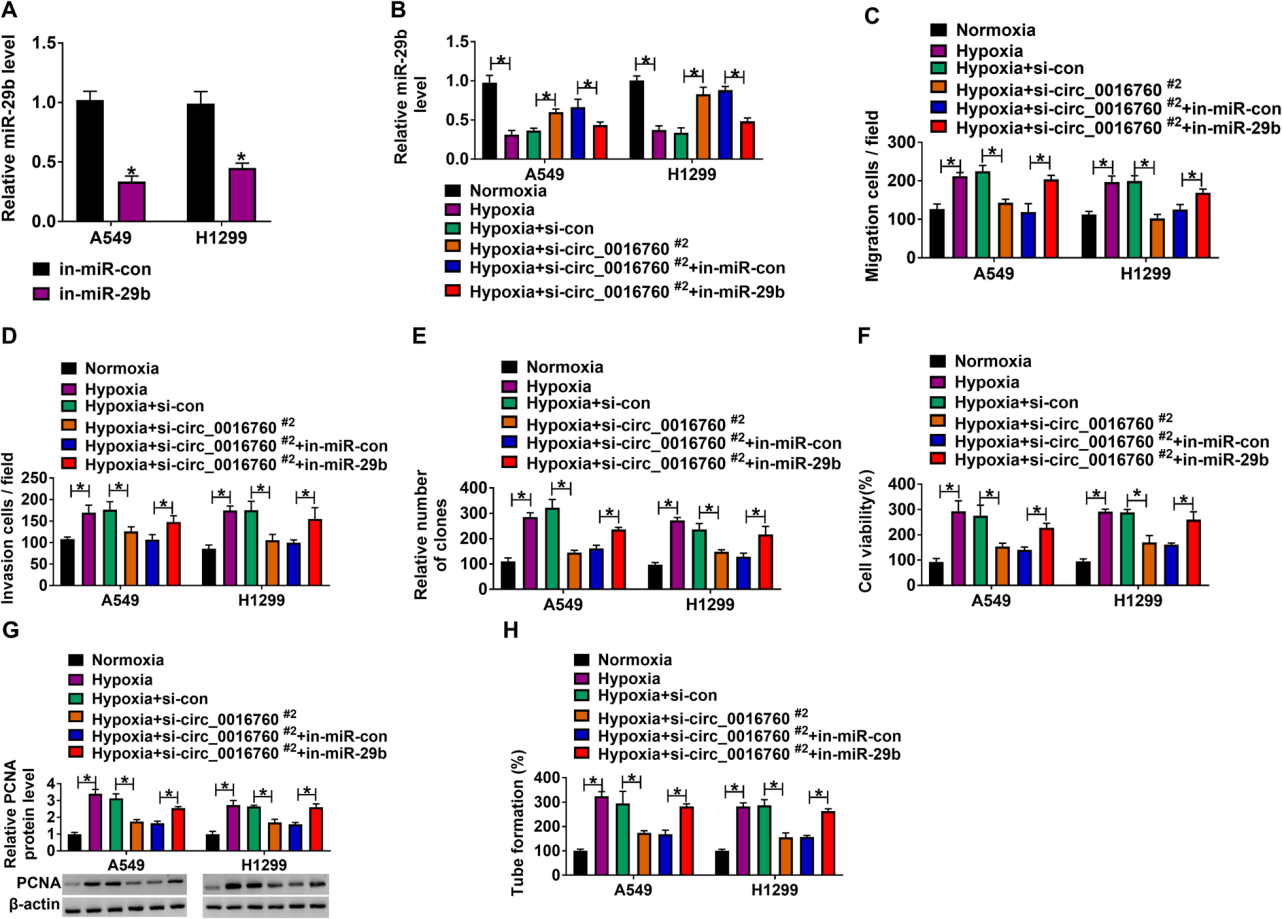
Circ_0016760 regulated NSCLC cell processes and angiogenic ability under hypoxia via sponging miR-29b. **A** MiR-29b expression was detected by qRT-PCR in A549 and H1299 cells transfected with in-miR-29b or in-miR-con. **B** The impacts between circ_0016760 knockdown and miR-29b inhibitor on miR-29b expression under hypoxia were demonstrated by qRT-PCR in A549 and H1299 cells. **C**, **D** Transwell assay was employed to unveil the effects between circ_0016760 silencing and miR-29b inhibitor on the migration and invasion of A549 and H1299 cells under hypoxia. **E**, **F** Cell colony formation and MTT assays were performed to reveal the impacts between circ_0016760 repression and miR-29b inhibitor on the proliferation of A549 and H1299 cells under hypoxia. **G** Western blot assay was employed to unveil the effects between circ_0016760 silencing and miR-29b inhibitor on PCNA expression under hypoxia. **H** Tube formation assay was employed to unveil the effects between circ_0016760 silencing and miR-29b inhibitor on the tube formation of A549 and H1299 cells under hypoxia. *P < 0.05

Given the binding relationship between circ_0016760 and miR-29b, whether circ_0016760 modulated NSCLC process by sponging miR-29b under hypoxia was further disclosed. Result first showed that miR-29b expression was downregulated after transfection with miR-29b inhibitor in A549 and H1299 cells (Fig. 5A), suggesting the success of miR-29b knockdown. Subsequently, we found that circ_0016760 silencing upregulated miR-29b expression under hypoxia, whereas miR-29b inhibitor reserved this impact (Fig. 5B). The data from Fig. 5C–F showed that circ_0016760 knockdown repressed the migration, invasion and proliferation of A549 and H1299 cells under hypoxia, which was restrained after transfection of miR-29b inhibitor. Additionally, circ_0016760 repression reduced PCNA expression and tube formation under hypoxia; however, miR-29b inhibitor impaired this influence (Fig. 5G, H). These findings demonstrated that circ_0016760 modulated NSCLC cell malignancy and angiogenesis under hypoxia through sponging miR-29b.

### MiR-29b repressed NSCLC cell processes and angiogenic ability by binding to HIF1A

**Fig. 6:**
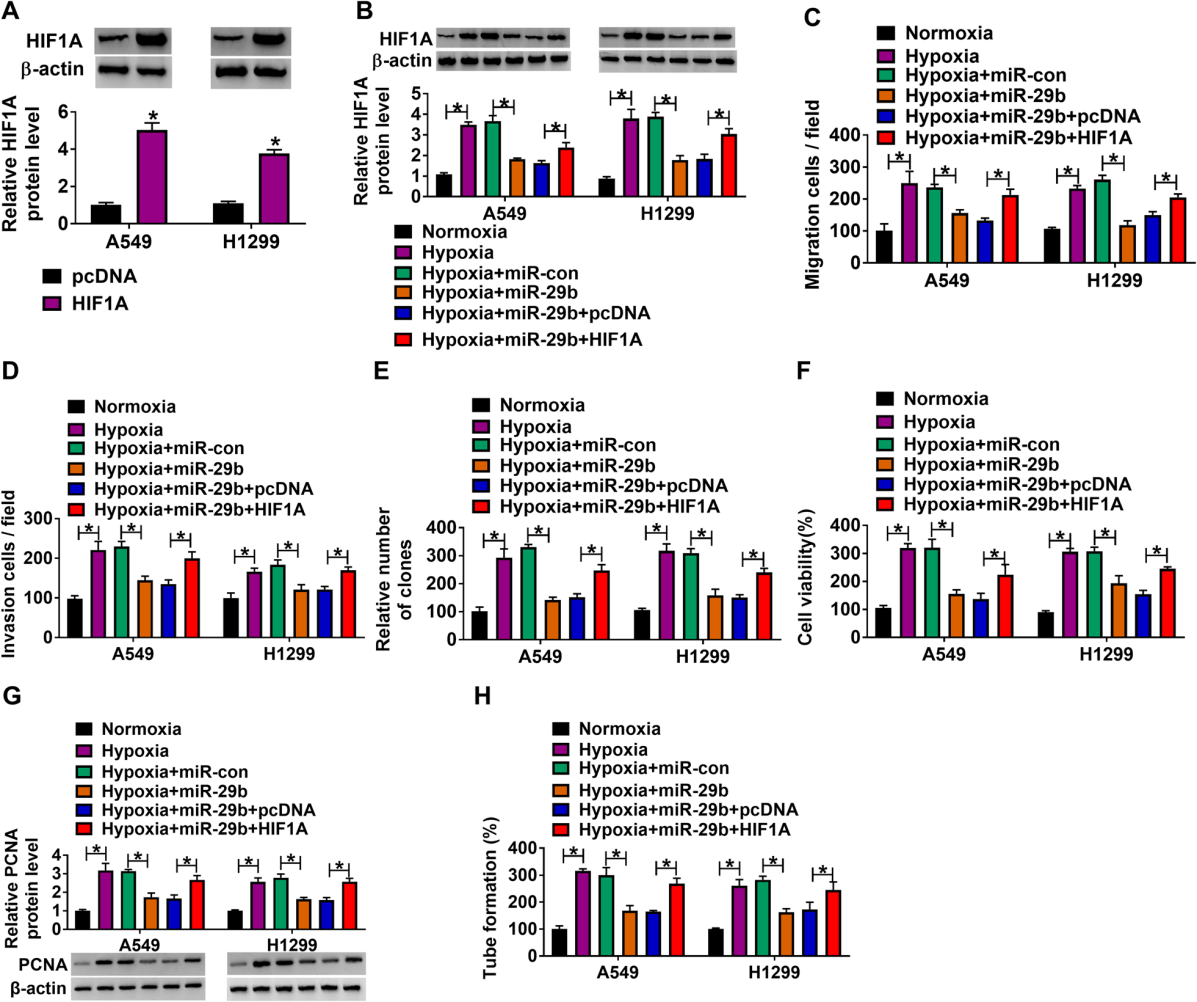
MiR-29b repressed NSCLC cell processes and angiogenic ability by binding to HIF1A. **A** The overexpression efficiency of HIF1A was detected by western blot in A549 and H1299 cells. **B** The effects between miR-29b and HIF1A on HIF1A protein expression under hypoxia were revealed by western blot in A549 and H1299 cells. **C**, **D** Transwell assay was performed to demonstrate the influences between miR-29b and HIF1A overexpression on the migration and invasion of A549 and H1299 cells under hypoxia. **E**, **F** The influences between miR-29b mimic and HIF1A overexpression on cell proliferation under hypoxia were unveiled by cell colony formation and MTT assays in A549 and H1299 cells. **G** The effects between miR-29b and HIF1A on PCNA protein expression under hypoxia were revealed by western blot in A549 and H1299 cells. **H** The effects between miR-29b and HIF1A on tube formation expression under hypoxia were revealed by tube formation assay in A549 and H1299 cells. *P < 0.05

Whether miR-29b regulated NSCLC process by binding to HIF1A after hypoxia treatment was disclosed in this part. Result initially presented the high efficiency of HIF1A in upregulating HIF1A expression (Fig. 6A). Subsequently, western blot assay presented that miR-29b overexpression dramatically decreased HIF1A protein expression under hypoxia, which was restored after HIF1A overexpression (Fig. 6B). The migration, invasion and proliferation of A549 and H1299 cells were repressed after transfection with miR-29b mimic under hypoxia, whereas HIF1A overexpression hindered these effects (Fig. 6C–F). Besides, the decreased expression of PCNA and tube formation by miR-29b overexpression under hypoxia were relieved by enforced HIF1A expression (Fig. 6G, H). Collectively, miR-29b regulated cell malignancy and tube formation by interacting with HIF1A under hypoxia in NSCLC cells.

### PESV treatment inhibited tumor formation by repressing circ_0016760 expression in vivo

**Fig. 7:**
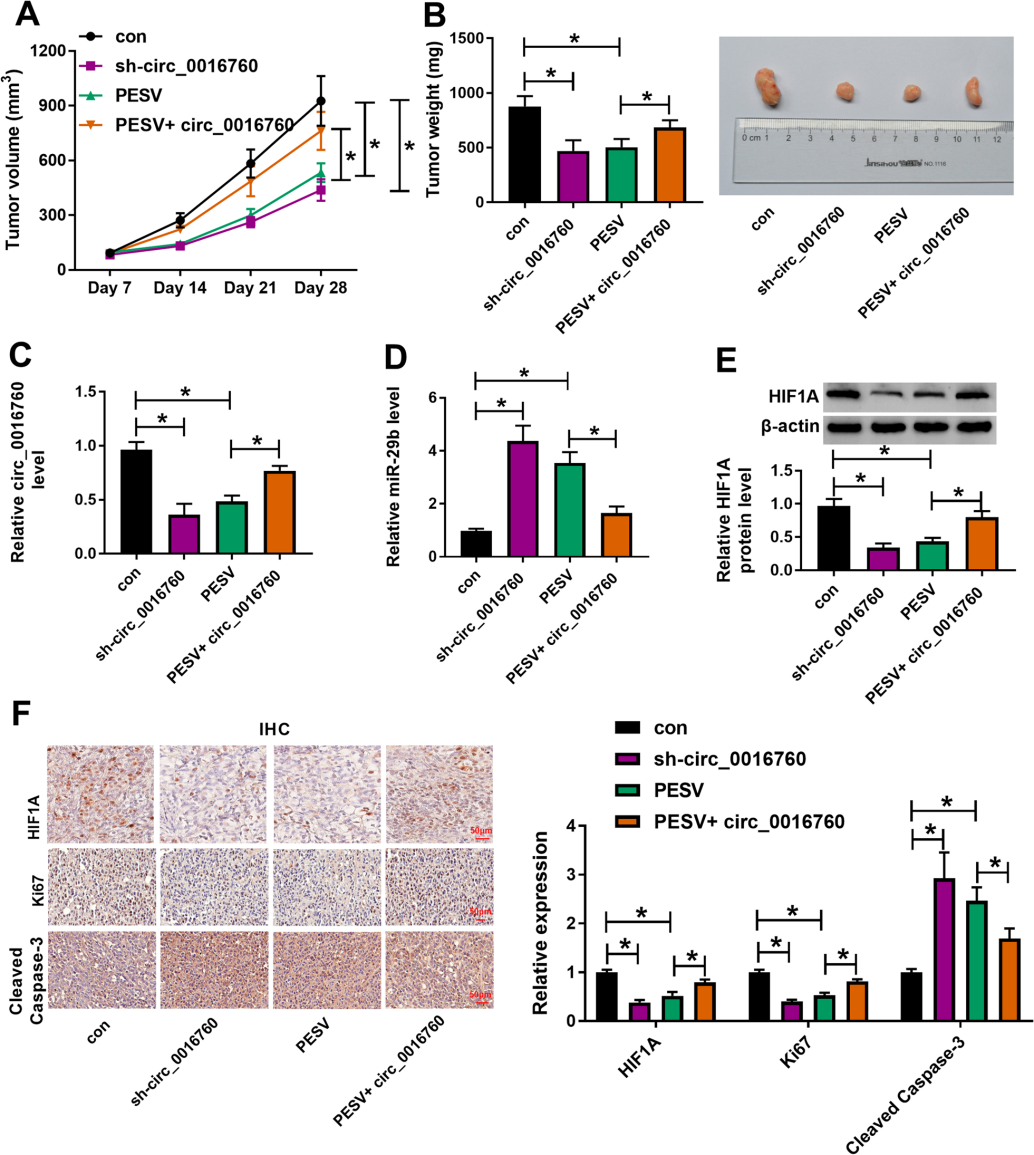
PESV regulated tumor formation by inhibiting circ_0016760 expression in vivo. **A**, **B** The impacts between PESV treatment and circ_0016760 on the volume and weight of tumors were disclosed in *vivo.* **C**, **D** QRT-PCR was performed to determine the influences between PESV treatment and circ_0016760 on the expression levels of circ_0016760 and miR-29b in vivo. **E**, **F** Western blot and IHC assays were conducted to demonstrate the effects between PESV treatment and circ_0016760 on HIF1A protein expression in vivo. *P < 0.05

The effects between PESV treatment and circ_0016760 on tumorigenesis in vivo were further revealed. Results showed that circ_0016760 silencing or PESV treatment inhibited the volume and weight of tumors, whereas circ_0016760 overexpression in combination with PESV reversed the inhibitory effects of PESV treatment on the volume and weight of tumors (Fig. 7A, B). Additionally, the combination treatment hindered PESV-mediated effects on the expression of circ_0016760 and HIF1A (Fig. 7C, E). Meanwhile, miR-29b expression was upregulated by PESV treatment; however, enforced circ_0016760 expression restrained PESV-induced effect (Fig. 7D). Additionally, the combination of PESV with circ_0016760 reversed PESV-induced dysregulated expression of HIF1A, Ki67 and Cleaved caspase-3 (Fig. 7F). All these data demonstrated that PESV treatment inhibited tumor formation via decreasing the expression of circ_0016760 and HIF1A and increasing miR-29b expression level in vivo.

## Discussion

Most of NSCLC patients are difficult to be cured by general treatment owing to late diagnosis [28]. For malignant cancers, chemotherapeutic drugs are less effective owing to their poor specificity, and lots of venoms specially targeting cellular elements lit a new hope for cancer therapy [29]. Previous researches disclosed that venom-derived peptides participated in regulating cell metastasis and growth of tumor [30, 31]. PESV, a polypeptide extracted from scorpion venom, has been found to reverse multi-drug resistance of leukemia stem cell [32]. In addition, PESV represses cell malignancy in prostate cancer [6], hepatocellular carcinoma [33] and lung cancer [7]. Nevertheless, the roles of PESV in NSCLC process under hypoxia are unknown. In this study, we demonstrated that PESV inhibited the malignant behaviors of NSCLC cells by regulating circ_0016760/miR-29b/HIF1A axis under hypoxia.

Herein, circ_0016760 expression was upregulated in NSCLC tissue and cell samples. Hypoxia treatment induced cell proliferation, migration, invasion and tube formation, while circ_0016760 silencing attenuated these effects, suggesting that circ_0016760 could promote NSCLC process. Li et al. also demonstrated that circ_0016760 expression was significantly augmented in NSCLC specimens and cells, and its upregulation facilitated cell proliferation and metastasis, while suppressed cell apoptosis [11, 34]. Hao et al. also disclosed that circ_0016760 knockdown repressed NSCLC cell colony-forming ability, growth and metastasis [12, 35]. These findings demonstrated that circ_0016760 acted as an oncogene in NSCLC. Our findings were consistent with these data. Beyond that, our data indicate the promoting effect of circ_0016760 on tube formation. Additionally, in this research, PESV treatment downregulated circ_0016760 expression. PESV exposure repressed cell proliferation, migration, invasion and angiogenesis after hypoxia treatment. Considering the above results, we hypothesized that PESV might repress NSCLC cell malignancy under hypoxia by regulating circ_0016760. In order to prove the hypothesis, rescue experiments were employed. Data showed that circ_0016760 overexpression relieved the inhibitory effects of PESV on NSCLC cell processes under hypoxia. In vivo tumor experiments indicated that enforced circ_0016760 expression attenuated the inhibitory effect of PESV on tumor formation. These results demonstrated that PESV could regulate NSCLC process by repressing circ_0016760 expression for the first time.

CircRNA commonly functions as a miRNA sponge to modulate the reciprocity of miRNA and protein [36]. Based on this theory, the underlying mechanism of circ_0016760 in mediating the effects of PESV on NSCLC process under hypoxia was unveiled. Results demonstrated that circ_0016760 was a sponge of miR-29b, and miR-29b targeted HIF1A. As reported, c-Myc facilitated cell growth and invasion by suppressing miR-29b in NSCLC [37]; miR-29b also inhibited cell migration and invasion in NSCLC [38]. HIF1A is an important transcription factor related to tumor cell survival, angiogenesis and invasion [39, 40]. Circ_0014130 was indicated to contribute to NSCLC cell proliferation and metastasis by inducing HIF1A production [41]; miR-130a hindered cell migration and invasion under hypoxia by binding to HIF1A in NSCLC [42]. In the work of Chang et al., we found that HIF1A regulated lung cancer progression through association with MMP9, a factor related to degradation of extracellular matrix and vascular remodeling [43]. Besides, previous data showed that HIF1A combined with the transcription factor of epithelial-to-mesenchymal transition, Snail, to mediate the development of lung cancer [44]. Based on these evidences, we hypothesized that circ_0016760 regulated NSCLC cell processes by upregulating HIF1A through sponging miR-29b. As a result, HIF1A expression was upregulated by circ_0016760 overexpression, whereas miR-29b mimic attenuated this effect in NSCLC cells treated with PESV or without. Further data suggested that miR-29b inhibitor restored circ_0016760 silencing-induced repression of NSCLC cell processes under hypoxia, and HIF1A overexpression impaired the repressing impacts of miR-29b mimic on NSCLC cell processes under hypoxia. These evidences demonstrated that circ_0016760 regulated NSCLC process by miR-29b/HIF1A pathway under hypoxia. Furthermore, in vivo data displayed that enforced circ_0016760 expression attenuated the decreased expression of circ_0016760 and HIF1A, and the increased expression of miR-29b expression induced by PESV treatment, implying that PESV could regulate miR-29b and HIF1A by inhibiting circ_0016760.

However, some shortcomings should be considered when evaluating our findings. Firstly, patient-derived tumor xenograft should be performed to verify the novel mechanism. Besides, the direct evidence between HIF1A and circ_0016760/miR-29b axis in regulating PESV-mediated NSCLC progression under hypoxia is lacking in the present study. Enhancer of zeste homolog 2 (EZH2) is the enzymatic subunit of polycomb-repressive complex 2 and contributes to transcriptional silencing in the development of lung cancer [45]. Previous data have showed that EZH2 regulates immune escape of lung cancer through HIF1A [46]. Thus, whether circ_0016760/miR-29b/HIF1A/EZH2 pathway is responsible for PESV-mediated NSCLC cell malignancy under hypoxia will be investigated in future.

## Conclusion

PESV treatment-mediated NSCLC progression under hypoxia involved the downregulation of circ_0016760. Circ_0016760 overexpression counteracted PESV-induced repression of NSCLC cell migration, invasion, and tube formation under hypoxia treatment. The inner mechanism was PESV treatment-induced downregulation of circ_0016760 expression activated miR-29b to inhibit HIF1A production. The flowchart of the mechanism responsible for PESV-mediated NSCLC progression was shown in Additional file 4: Fig. S3. Our findings open up a new horizon for further investigation on the therapy of NSCLC with PESV. Besides, the study suggests the possibility of PESV in combination with circ_0016760 depletion as a therapeutic agent for NSCLC.

## Supplementary Information

**Additional file 1: Table S1.** The clinicopathologic features of 43 NSCLC patients.

**Additional file 2: Figure S1. **The effect of PESV treatment on the apoptosis of A549, H1299 and BEAS-2B cells was determined by flow cytometry analysis. *P < 0.05.

**Additional file 3: Figure S2. **The effects between ectopic circ_0016760 expression and miR-29b mimic on miR-29b expression and HIF1A protein expression were determined by qRT-PCR and western blot, respectively, in A549 and H1299 cells treated with PESV. *P < 0.05.

**Additional file 4: Figure S3.** The flowchart of the mechanism responsible for PESV-mediated NSCLC progression.

## Data Availability

Not applicable.

## References

[1] Bray F, Ferlay J, Soerjomataram I, Siegel RL, Torre LA, Jemal A (2018). Global cancer statistics 2018: GLOBOCAN estimates of incidence and mortality worldwide for 36 cancers in 185 countries. CA Cancer J Clin.

[2] Tsim S, O'Dowd CA, Milroy R, Davidson S (2010). Staging of non-small cell lung cancer (NSCLC): a review. Respir Med.

[3] Liu G, Pei F, Yang F, Li L, Amin AD, Liu S (2017). Role of autophagy and apoptosis in non-small-cell lung cancer. Int J Mol Sci.

[4] Rey S, Schito L, Wouters BG, Eliasof S, Kerbel RS (2017). Targeting hypoxia-inducible factors for antiangiogenic cancer therapy. Trends Cancer.

[5] Huang R, Zong X (2017). Aberrant cancer metabolism in epithelial-mesenchymal transition and cancer metastasis: Mechanisms in cancer progression. Crit Rev Oncol Hematol.

[6] Zhang YY, Wu LC, Wang ZP, Wang ZX, Jia Q, Jiang GS (2009). Anti-proliferation effect of polypeptide extracted from scorpion venom on human prostate cancer cells in vitro. J Clin Med Res.

[7] Wang X, Wang Z, Zhang Y, Jia Q, Wang Z, Zhang J (2012). Mechanisms for inhibition effects of polypeptide extract from scorpion venom (PESV) on proliferation of A549 cell lines in vitro. Zhongguo Zhong Yao Za Zhi.

[8] Shen B, Wang Z, Li Z, Song H, Ding X (2019). Circular RNAs: an emerging landscape in tumor metastasis. Am J Cancer Res.

[9] Gao P, Wang Z, Hu Z, Jiao X, Yao Y (2020). Circular RNA circ_0074027 indicates a poor prognosis for NSCLC patients and modulates cell proliferation, apoptosis, and invasion via miR-185-3p mediated BRD4/MADD activation. J Cell Biochem.

[10] Chen T, Yang Z, Liu C, Wang L, Yang J, Chen L (2019). Circ_0078767 suppresses non-small-cell lung cancer by protecting RASSF1A expression via sponging miR-330-3p. Cell Prolif.

[11] Li Y, Hu J, Li L, Cai S, Zhang H, Zhu X (2018). Upregulated circular RNA circ_0016760 indicates unfavorable prognosis in NSCLC and promotes cell progression through miR-1287/GAGE1 axis. Biochem Biophys Res Commun.

[12] Hao Y, Xi J, Peng Y, Bian B, Hao G, Xi Y (2020). Circular RNA Circ_0016760 modulates non-small-cell lung cancer growth through the miR-577/ZBTB7A axis. Cancer Manag Res.

[13] Langsch S, Baumgartner U, Haemmig S, Schlup C, Schäfer SC, Berezowska S (2016). miR-29b mediates NF-κB Signaling in KRAS-induced non-small cell lung cancers. Cancer Res.

[14] Di Leva G, Garofalo M, Croce CM (2014). MicroRNAs in cancer. Annu Rev Pathol.

[15] Yan B, Guo Q, Fu FJ, Wang Z, Yin Z, Wei YB (2015). The role of miR-29b in cancer: regulation, function, and signaling. Onco Targets Ther.

[16] Xie Y, Zhao F, Zhang P, Duan P, Shen Y (2020). miR-29b inhibits non-small cell lung cancer progression by targeting STRN4. Hum Cell.

[17] Wang H, Guan X, Tu Y, Zheng S, Long J, Li S (2015). MicroRNA-29b attenuates non-small cell lung cancer metastasis by targeting matrix metalloproteinase 2 and PTEN. J Exp Clin Cancer Res.

[18] Semenza GL (1998). Hypoxia-inducible factor 1: master regulator of O2 homeostasis. Curr Opin Genet Dev.

[19] Schito L, Semenza GL (2016). Hypoxia-inducible factors: master regulators of cancer progression. Trends Cancer.

[20] Semenza GL (1985). HIF-1: mediator of physiological and pathophysiological responses to hypoxia. J Appl Physiol.

[21] Liu Y, Nie H, Zhang K, Ma D, Yang G, Zheng Z (2014). A feedback regulatory loop between HIF-1α and miR-21 in response to hypoxia in cardiomyocytes. FEBS Lett.

[22] Ding G, Huang G, Liu HD, Liang HX, Ni YF, Ding ZH (2013). MiR-199a suppresses the hypoxia-induced proliferation of non-small cell lung cancer cells through targeting HIF1α. Mol Cell Biochem.

[23] Wang M, Wang W, Wang J, Zhang J (2018). MiR-182 promotes glucose metabolism by upregulating hypoxia-inducible factor 1α in NSCLC cells. Biochem Biophys Res Commun.

[24] He Q, Zhao L, Liu X, Zheng J, Liu Y, Liu L (2019). MOV10 binding circ-DICER1 regulates the angiogenesis of glioma via miR-103a-3p/miR-382-5p mediated ZIC4 expression change. J Exp Clin Cancer Res.

[25] Xu P, Jia S, Wang K, Fan Z, Zheng H, Lv J (2020). MiR-140 inhibits classical swine fever virus replication by targeting Rab25 in swine umbilical vein endothelial cells. Virulence.

[26] Jin T, Liu M, Liu Y, Li Y, Xu Z, He H (2020). Lcn2-derived circular RNA (hsa_circ_0088732) inhibits cell apoptosis and promotes EMT in glioma via the miR-661/RAB3D axis. Front Oncol.

[27] Huang D-W, Huang M, Lin X-S, Huang Q (2017). CD155 expression and its correlation with clinicopathologic characteristics, angiogenesis, and prognosis in human cholangiocarcinoma. Onco Targets Ther.

[28] Duma N, Santana-Davila R, Molina JR (2019). Non-small cell lung cancer: epidemiology, screening, diagnosis, and treatment. Mayo Clin Proc.

[29] Lewis RJ, Garcia ML (2003). Therapeutic potential of venom peptides. Nat Rev Drug Discov.

[30] Soroceanu L, Manning TJ, Sontheimer H (1999). Modulation of glioma cell migration and invasion using Cl(-) and K(+) ion channel blockers. J Neurosci.

[31] Fu YJ, Yin LT, Liang AH, Zhang CF, Wang W, Chai BF (2007). Therapeutic potential of chlorotoxin-like neurotoxin from the Chinese scorpion for human gliomas. Neurosci Lett.

[32] Yang XD, Wang XL, Yang WH, Zhao L, Yan TG (2016). Research of reversal effect of PESV to multi-drug resistance of leukemia stem cell. Zhongguo Zhong Yao Za Zhi.

[33] Chen H, Zhidan W, Xia R, Zhaoxia W, Qing J, Qiang G (2016). Scorpion venom activates natural killer cells in hepatocellular carcinoma via the NKG2D-MICA pathway. Int Immunopharmacol.

[34] Zhu Z, Wu Q, Zhang M, Tong J, Zhong B, Yuan K (2021). Hsa_circ_0016760 exacerbates the malignant development of non-small cell lung cancer by sponging miR-145-5p/FGF5. Oncol Rep.

[35] Yan X, Wang T, Wang J (2020). Circ_0016760 acts as a sponge of MicroRNA-4295 to enhance E2F Transcription Factor 3 Expression And Facilitates Cell Proliferation And Glycolysis In Nonsmall Cell Lung Cancer. Cancer Biother Radiopharm.

[36] Rybak-Wolf A, Stottmeister C, Glažar P, Jens M, Pino N, Giusti S (2015). Circular RNAs in the mammalian brain are highly abundant, conserved, and dynamically expressed. Mol Cell.

[37] Wu DW, Hsu NY, Wang YC, Lee MC, Cheng YW, Chen CY (2015). c-Myc suppresses microRNA-29b to promote tumor aggressiveness and poor outcomes in non-small cell lung cancer by targeting FHIT. Oncogene.

[38] Wang HY, Tu YS, Long J, Zhang HQ, Qi CL, Xie XB (2015). SRF-miR-29b-MMP2 axis inhibits NSCLC invasion and metastasis. Int J Oncol.

[39] Huang Y, Lin D, Taniguchi CM (2017). Hypoxia inducible factor (HIF) in the tumor microenvironment: friend or foe?. Sci China Life Sci.

[40] Wohlkoenig C, Leithner K, Deutsch A, Hrzenjak A, Olschewski A, Olschewski H (2011). Hypoxia-induced cisplatin resistance is reversible and growth rate independent in lung cancer cells. Cancer Lett.

[41] Chi Y, Luo Q, Song Y, Yang F, Wang Y, Jin M (2019). Circular RNA circPIP5K1A promotes non-small cell lung cancer proliferation and metastasis through miR-600/HIF-1α regulation. J Cell Biochem.

[42] Shi J, Wang H, Feng W, Huang S, An J, Qiu Y (2020). MicroRNA-130a targeting hypoxia-inducible factor 1 alpha suppresses cell metastasis and Warburg effect of NSCLC cells under hypoxia. Life Sci.

[43] Chang Y, Chan Y, Chang W, Lin Y, Yang C, Su C (2017). Feedback regulation of ALDOA activates the HIF-1α/MMP9 axis to promote lung cancer progression. Cancer Lett.

[44] Park J, Moon M, Kim J, Oh S (2021). TOPK mediates hypoxia-induced epithelial-mesenchymal transition and the invasion of nonsmall-cell lung cancer cells via the HIF-1α/snail axis. Biochem Biophys Res Commun.

[45] Su M, Xiao Y, Tang J, Wu J, Ma J, Tian B (2018). Role of lncRNA and EZH2 interaction/regulatory network in lung cancer. J Cancer.

[46] Zhao Y, Wang XX, Wu W, Long H, Huang J, Wang Z (2019). EZH2 regulates PD-L1 expression via HIF-1α in non-small cell lung cancer cells. Biochem Biophys Res Commun.

