# Supplementary material for: PESV represses non-small cell lung cancer cell malignancy through circ_0016760 under hypoxia
Source: Cancer Cell Int. 2021 Nov 27;21:628. doi: 10.1186/s12935-021-02336-6 (PMC8626912; doi:10.1186/s12935-021-02336-6)
Supplement: Supplementary file 1 — Additional file 1: Table S1. The clinicopathologic features of 43 NSCLC patients. [file 12935_2021_2336_MOESM1_ESM.docx]

**Table S1 The clinicopathologic features of 43 NSCLC patients**

| Clinicopathologic features | N | % |
| --- | --- | --- |
| Age(years) | | |
| ≥55 | 19 | 44.2 |
| <55 | 24 | 55.8 |
| Gender | | |
| Male | 27 | 62.8 |
| Female | 16 | 37.2 |
| TNM stage | | |
| Ⅰ+Ⅱ | 34 | 79.1 |
| Ⅲ | 9 | 20.9 |
| Tumor size (cm) | | |
| ≥3 | 11 | 25.6 |
| <3 | 32 | 74.4 |
