# Supplementary figures and images for: PESV represses non-small cell lung cancer cell malignancy through circ_0016760 under hypoxia
Source: Cancer Cell Int. 2021 Nov 27;21:628. doi: 10.1186/s12935-021-02336-6 (PMC8626912; doi:10.1186/s12935-021-02336-6)

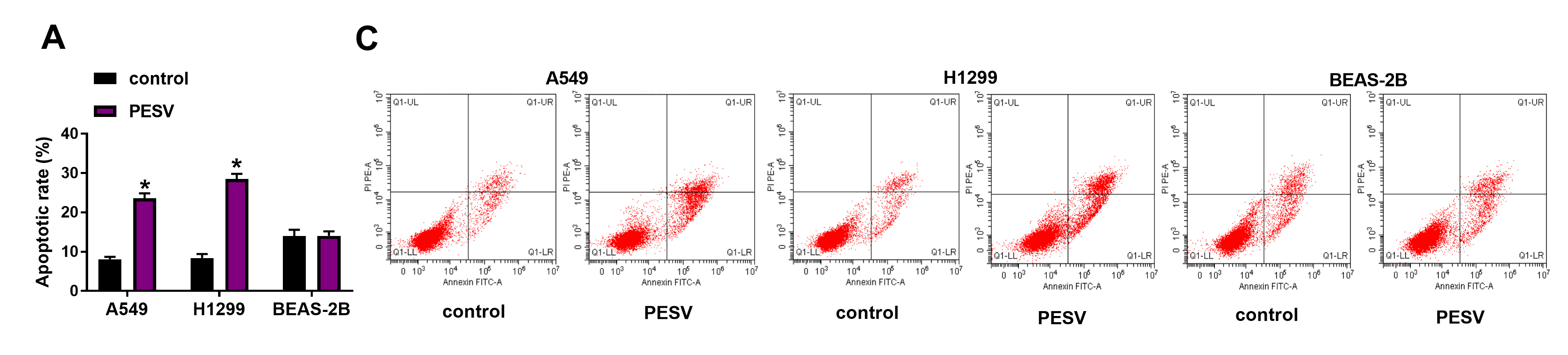

Supplement: Supplementary file 2 — Additional file 2: Figure S1. The effect of PESV treatment on the apoptosis of A549, H1299 and BEAS-2B cells was determined by flow cytometry analysis. *P < 0.05. [file 12935_2021_2336_MOESM2_ESM.tif]

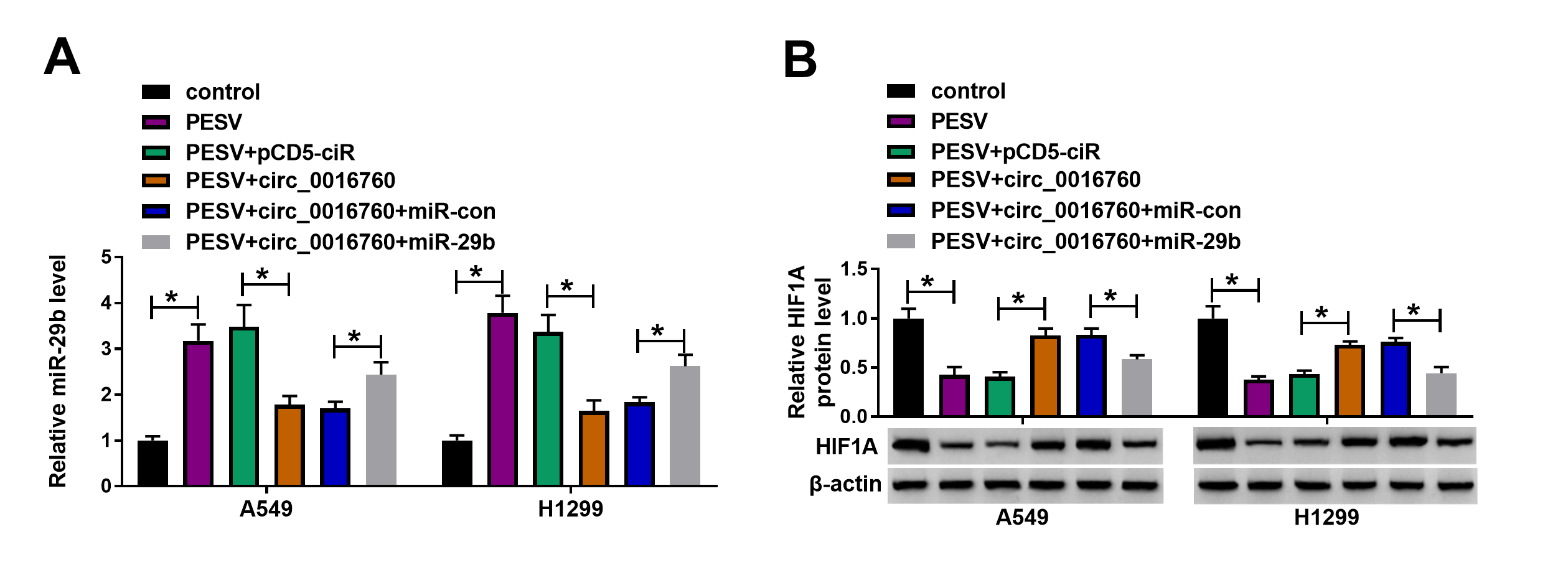

Supplement: Supplementary file 3 — Additional file 3: Figure S2. The effects between ectopic circ_0016760 expression and miR-29b mimic on miR-29b expression and HIF1A protein expression were determined by qRT-PCR and western blot, respectively, in A549 and H1299 cells treated with PESV. *P < 0.05. [file 12935_2021_2336_MOESM3_ESM.tif]

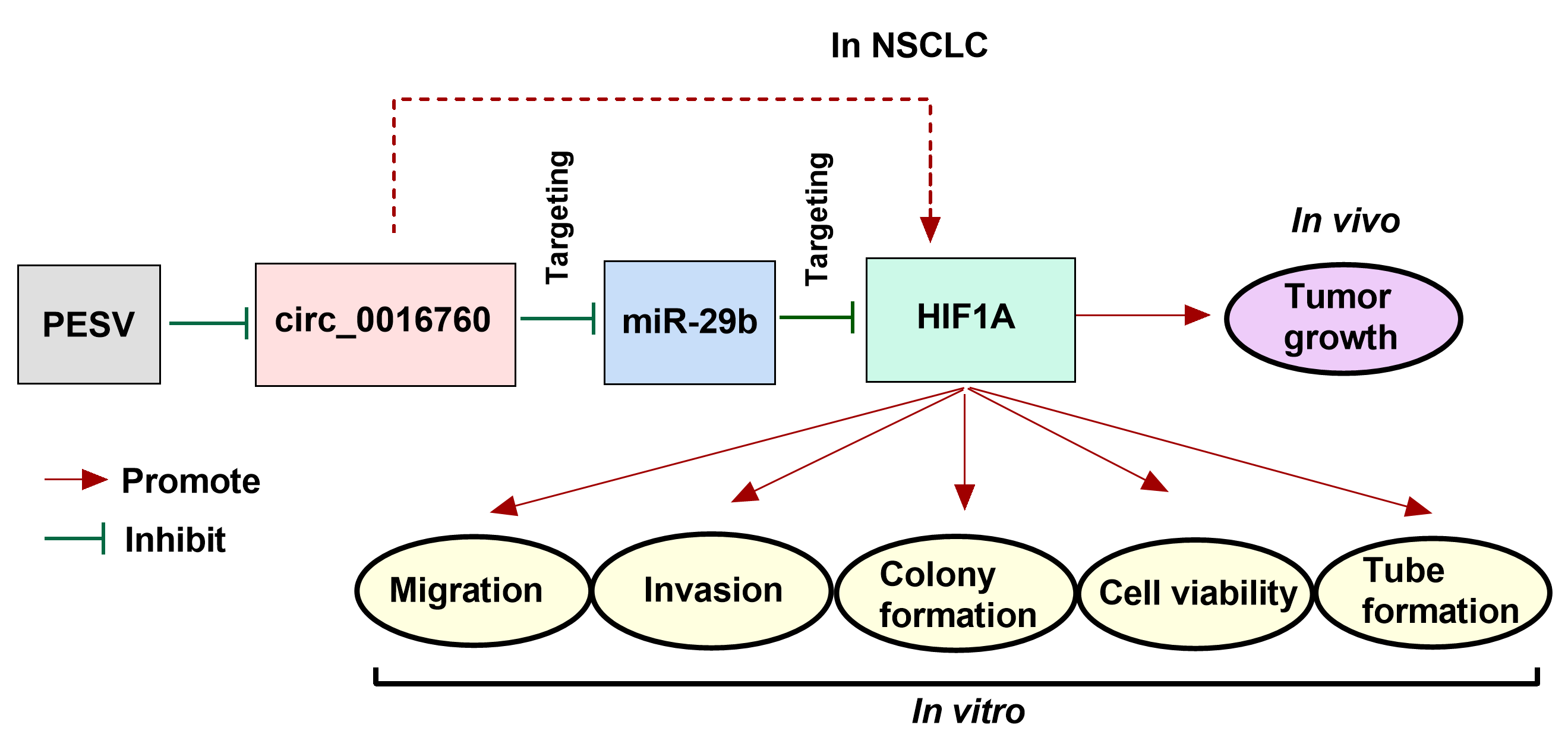

Supplement: Supplementary file 4 — Additional file 4: Figure S3. The flowchart of the mechanism responsible for PESV-mediated NSCLC progression. [file 12935_2021_2336_MOESM4_ESM.tif]
